# Quantifying American Alligator (*Alligator mississippiensis*) Nest Depredation by Wild Pigs (*Sus scrofa*)

**DOI:** 10.3390/ani16213408

**Published:** 2026-10-29

**Authors:** Alyssa C. Freeman, Jonathan K. Warner, Justine L. Smith, John M. Tomeček

**Affiliations:** 1 Department of Rangeland, Wildlife, and Fisheries Management, Texas A&M University, College Station, TX 77843, USA; 2 Texas Parks and Wildlife Department, Austin, TX 78744, USA

**Keywords:** alligator nest depredation, invasive species, nest predation, principal component analysis, wildlife damage management, wildlife management

## Abstract

Understanding interactions between native and invasive species is crucial for effective wildlife management, as predation by invasive species can significantly impact native populations. This study investigated the factors influencing wild pig (*Sus scrofa*) predation on 28 American alligator (*Alligator mississippiensis*) nests in Texas coastal marshes between 2022 and 2023. We examined variables including nest location, wetland type, soil type, landcover, distance to roads, distance to management headquarters (i.e., human infrastructure), distance to levees, distance to trees, and salinity levels to determine their relationship with nest predation. We analyzed data using generalized linear regression models and evaluated the models’ performance using AICc values and the Hosmer–Lemeshow goodness-of-fit test. Our results revealed that proximity to roads was negatively associated with nest predation risk (β = −0.01, *p* = 0.05), while increased distance from management headquarters was positively correlated with predation (β = 0.0004, *p* = 0.09). This study’s findings indicate that distance to roads influence predation dynamics, with potential implications for wildlife managers seeking to mitigate wild pig depredation of alligator nests and potentially other ground-nesting reptiles and birds. These insights can guide habitat management strategies and inform conservation efforts by identifying high-risk areas and enhancing protective measures for alligator nests.

## Simple Summary

This study investigated the factors that influenced wild pigs (*Sus scrofa*), an invasive species in the United States, to predate on 28 American alligator (*Alligator mississippiensis*), a native species that was historically listed as endangered, nests. Wild pigs will often forage on other ground-nesting reptile and bird nests and have been briefly explored in the scientific literature. In this study, we find that human-related metrics, such as distance to roads and distance to buildings, have influenced the potential for wild pig predation on alligator nests. Almost all nests (67.9%) were visited by wild pigs, with most (61%) nests being foraged by wild pigs. Nests that were closer to roads were more likely to be predated by wild pigs whereas nests closer to buildings were not. The influence of human infrastructure on predation risk is an important factor for wildlife managers to account for regarding habitat management and wild pig damage reduction.

## 1. Introduction

Wild pigs (*Sus scrofa*) are the most widely distributed ungulate in the world [1,2]. Wild pigs were introduced to North America for the first time as a food source for Spanish colonists in the mid-1500s [3,4,5]. Wild pigs have been introduced to the United States on multiple occasions throughout history and their release into the wild has been accidental as well as intentional [6]. According to the United States Department of Agriculture’s National Invasive Species Information Center, wild pigs occur in at least 35 states, and their population is estimated to be over 6 million. Of those wild pigs, at least 2.6 million are estimated to reside in Texas, where they occur in 252 out of 254 counties [7,8]. Wild pigs exhibit high reproductive capacity and early maturation, making population control challenging once they become established [9,10,11,12]. Their spread is facilitated by rapid population growth, few predators, a remarkable ability to adapt swiftly to new environments, and human transport to new areas [1,2]. Wild pigs thrive in a diverse array of habitats, feeding opportunistically on both plant and animal matter.

Wild pig diet plasticity is another contributing factor to their widespread success as an invasive species. Wild pigs are generalists, as well as opportunistic omnivores, where their diet consists of a wide variety of plants and animals available to them [2,6,13]. Part of their eating habit is to forage for food by overturning soil and vegetation, a process referred to as rooting. Rooting can cause direct damage to the vegetation as well as alter the soil nutrient cycling, cause soil erosion, and increase nutrient concentration in nearby water [2,6,14]. Wetland ecosystems in particular can be severely damaged by contamination caused by rooting [15].

Although plant matter makes up most of their diet, wild pigs have been documented to consume a variety of species, including insects, mammals, birds, amphibians, and reptiles [2,6,13,16,17]. While a wide range of fauna has been detected in wild pig stomach contents, it is unclear if it is due to active hunting or carrion consumption [2,6,18,19,20,21]. Wild pigs can be especially harmful to ground nesting avifauna and herpetofauna through nest depredation [13,16,17,22]. Although this is known, extensive documentation of nest predation on herpetofauna is limited.

An example of a nesting herpetofauna is the American alligator (*Alligator mississippiensis*, hereafter alligator) originally listed in 1967 as endangered under the Endangered Species Preservation Act, a precursor to the Endangered Species Act of 1973, and was successfully delisted and deemed fully recovered in 1987 [23]. The American alligator, native to North America largely persists between Florida and Texas, with ranges difficult to identify due to prior conservation concern [24]; population declines were largely due to commercial overharvesting for alligator hide for the fashion industry and habitat loss [25]. Nevertheless, even recovered species must be diligently observed to ensure their conservation continues amid changing environments.

The alligator breeding period includes courtship and copulation, while the nesting period includes nest construction, egg laying, incubation, and finally hatching. The onset of the breeding period can vary across their distribution, dependent on ambient temperatures, but typically begins in late winter or early spring (April), with egg laying typically occurring late spring or early summer (between May and July), and subsequent hatchling liberation approximately two months after laying [26]. Nest predation often occurs right before hatchling liberation [26]. Wild pigs, as nest predators [13,16,17,22], present a risk to alligator conservation, where concern has grown over the last few decades as wild pig populations continue to increase and expand. Although the range of the alligator completely overlaps with that of wild pigs in the United States, the interactions between these two species have not been well-examined.

Numerous studies have documented alligator nest predation by nonnative species such as exotic tegu (*Tupinambis merianae* [27]) and native species, including raccoons (*Procyon lotor* [26,28,29,30,31]), black bears (*Ursus americanus*, [30,32]), river otters (*Lontra canadensis* [31]), and more recently, Virginia opossums (*Didelphis virginiana* [33,34]) and bobcats (*Lynx rufus* [35]). Studies have mentioned alligator nest depredation by wild pigs, but to our knowledge there has not been any rigorous investigation of the pattern and process of these events [36,37,38,39,40,41,42]. One would expect alligators to be adapted to nest predation by native species, but that of exotic animals presents an additive source of mortality that is poorly understood. Understanding the nuances of the interactions between wild pigs and alligators is crucial for maintaining alligator conservation efforts and for managing the impacts of invasive wild pigs. This knowledge will help develop strategies to protect alligator nests and mitigate the broader ecological impacts of wild pigs on wetland ecosystems. This study quantifies nest predation by wild pigs on alligator nests on the upper coast of Texas and provides some insights into the potential scope and scale of wild pig impacts on alligator conservation. Specifically, we investigated landscape factors that influence wild pig predation on alligator nests across two nesting seasons in 2022 and 2023.

## 2. Materials and Methods

### 2.1. Study Site

**Figure 1:**
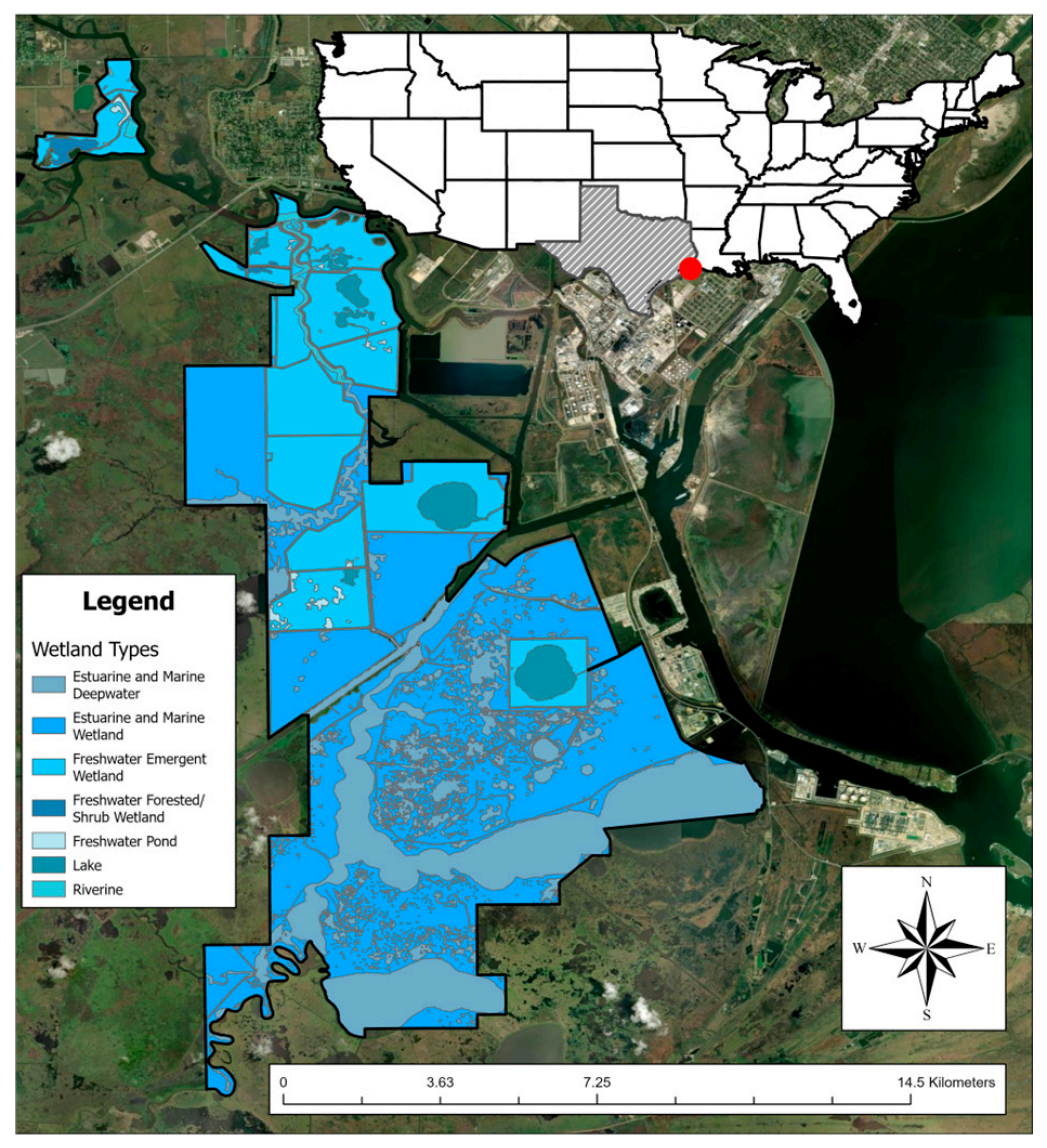
Map of the types of wetlands present at the J.D. Murphree Wildlife Management Area in Jefferson County, Texas. Map of the United States (upper right) indicates the state of Texas (grey shading) and location of the J.D. Murphree Wildlife Management Area (red dot).

We conducted our study on the J.D. Murphree Wildlife Management Area (hereafter Murphree WMA), a conservation site situated on the upper Texas coast in Jefferson County, managed by the Texas Parks and Wildlife Department (hereafter TPWD). Muphree WMA is one of the earliest alligator restoration site locations in Texas, hosting a high density of American alligators, and maintains robust and historical datasets of alligator populations, nest locations, and landscape characteristics [43]. Spanning 10,462 hectares, Murphree WMA included a range of wetland environments typical of the Texas Chenier Plain of the upper Texas coast, including freshwater to intermediate, brackish, and saline wetlands (Figure 1). Cattails (*Typha latifolia*), California bulrush (*Schoenoplectus californicus*), Jamaican sawgrass (*Cladium jamaicense*), lotus (*Nelumbo lutea*), and arrowheads (*Sagittaria* spp.) commonly grow in freshwater wetlands. Low salinity marshes (i.e., palustrine) house seashore paspalum (*Paspalum vaginatum*), cordgrass (*Spartina* spp.), banana waterlily (*Nymphaea mexicana*) and spike rushes (*Eleocharis palustris*). High salinity (brackish) marshes often contain cordgrass, salt grass (*Distichlis spicata*), widgeon grass (*Ruppia maritima*), and dwarf spike rush (*E. parvula*). Finally, salt marshes (i.e., estuarine) are dominated by oyster grass (*S. alterniflora*) [43].

### 2.2. Field Data Collection

We placed Reconyx Hyperfire and Reconyx Hyperfire II motion-triggered cameras (Reconyx, Holmen, WI, USA) at 28 active alligator nests according to locations geospatially marked during TPWD’s annual aerial nest surveys in July 2022 and 2023. In 2022, we set the cameras on 10 alligator nests between 4 and 5 August, then retrieved the cameras on 19 September. In 2023, we set the cameras on 18 alligator nests on 11 July and retrieved them on 28 September (*n* = 28). We defined the nest location as either a levee, bank, or open marsh based on feedback from the TPWD annual aerial survey and physical assessment while visiting each nest. We placed two cameras, secured to two 1.7 m steel T-posts facing one another, at each alligator nest to allow comprehensive documentation and reduce the capture of surrounding vegetation within the view field of the camera [44]. In addition, we did not remove vegetation surrounding the nests to minimize human disturbance in the area, nor did we visit the cameras again until the end of this study. Although a clear view of the nest was achieved with each camera, we did not set a specific height at which each camera was set to allow flexibility in locating T-posts such that nests were at the optimal location in the frame of the images. We programmed cameras to motion trigger at high sensitivity and collect three images in a rapid-fire burst with a 1 min delay between bursts. We processed and organized the images using Camelot 1.6.16 [45], an image managing software and augmented our detection accuracy using MegaDetector (MDv4.1), an open-source detection model [46]. We employed MegaDetector as an additional verification tool to minimize the risk of oversight in animal detections. We constructed a relational database in Filemaker Pro 19.6.3 (FileMaker, Inc., Santa Clara, CA, USA) to store data collected from the images. We defined nest success by the presence of footage indicating hatchling liberation and nest depredation as the detection of another species foraging through the nests (e.g., wild pig head position in the nest and perpendicular to the ground or raccoon paws interacting with the nest).

### 2.3. Remote Data Collection

Salinity impacts vegetation structure, which may indirectly influence wild pig foraging behavior [47,48]. Wild pigs have been documented to utilize salt marshes in search of roots, tubers, and invertebrates [49,50]. Researchers documented that alligator nesting density and success negatively correlates with salinity and positively correlates with freshwater levels [26,51,52]. Therefore, we used mean salinity in parts per thousand (ppt) from water samples collected by TPWD staff across the Murphree WMA. We calculated the average summer salinity values from nearby collection sites, collected monthly, that correlated with each monitored alligator nest, and assumed these values were representative of the nesting season.

In addition, we were interested in landscape characteristics and their influences on wild pig nest predation. We were interested in investigating the potential influence of human presence on depredation events by including distance to roads (i.e., unpaved grass or dirt roads) and distance to human infrastructure (hereafter referred to as headquarters) in meters. Since typical predators of alligator nests are arboreal (e.g., raccoons) we included distance to trees, in meters, to account for predation risk outside of wild pigs on alligator nests. Finally, we were interested in landscape metrics, such as soil type, landcover type, wetland type, and distance to levees, in meters, and their influence on predation risk. We extracted soil type from the USDA Natural Resources Conservation Service Web Soil Survey [53] and wetland type from the U.S. Fish and Wildlife Service from the National Wetlands Inventory [54]. For landcover type, we extracted data from the Coastal Change Analysis Program (C-CAP) [55]. Given the high resolution of the C-CAP raster data (range of 1–2.4 m/pixel depending on the geographic location [56]), we calculated the Euclidean distance from each alligator nest to the nearest road, levee, tree, and headquarters in meters. We conducted all spatial analyses in ArcMap 10.8.1 [56].

### 2.4. Statistical Analyses

We conducted analyses using RStudio version 4.6.1 [57] to examine factors affecting alligator nest predation by wild pigs. We ran a series of binary logistic regression models, with the response variable being nest predation (1 = nests that were predated and 0 = nests that were not), using the ‘stats’ package version 4.6.1 [57]. We created models that included following one or more of the following predictors: nest location (levee, bank, and open marsh), wetland type (riverine, estuarine and marine, and freshwater emergent), mean summer salinity (ppt), soil type (Leerco muck [LvA], Caplen mucky peat [CeA], Barnett mucky peat [BcA], and Bancker mucky peat [BaA]), landcover (palustrine scrub/shrub, palustrine emergent, estuarine scrub/shrub, estuarine emergent, barren land, and open water), distance to roads (m), distance to levees (m), distance to trees (m), and distance to headquarters (m). We performed a principal component analysis (hereafter PCA) with standardized continuous variables to account for potential multicollinearity between continuous predictor variables (i.e., mean summer salinity and distance to roads, levees, trees, and headquarters) and to control for variation at each alligator nesting site [58]. We assessed the performance of each assembled model using Akaike Information Criterion corrected for small sample sizes (AICc) and Akaike weights to compare model fit using the ‘MuMIn’ package version 1.48.19 [59]. To assemble our models, we utilized forward selection and backwards elimination for our predictor variables by using the ‘step’ function in the ‘stats’ package version 4.6.1 [57]. For forward selection, we began with a null model and added singular predictor variables (forward selection) to the null model. Predictor variables that do not improve upon the model (i.e., reduction in AICc) are thus excluded from the model building process. Conversely, a global model is built, and if removal of predictor variables does not improve upon the model (backwards elimination), then those predictor variables are retained [60]. We repeated these methods for global models with all predictor variables, and the global model with all predictor variables including the PCA analysis. Lastly, we identified the top performing model among all assembled models via AICc and assessed the associated goodness-of-fit using the Hosmer–Lemeshow test, which assesses the goodness-of-fit of the model by comparing observed and expected frequencies of predation events across different groups [61], using the ‘ResourceSelection’ package version 0.3-6 [62]. We considered models to be competing with <2 ΔAIC [63]. We interpreted model goodness-of-fit where model statistics and associated *p*-values > 0.05 indicated support for model fitness [61,64].

## 3. Results

### 3.1. Species Diversity and Depredation Rates at Alligator Nests

**Figure 2:**
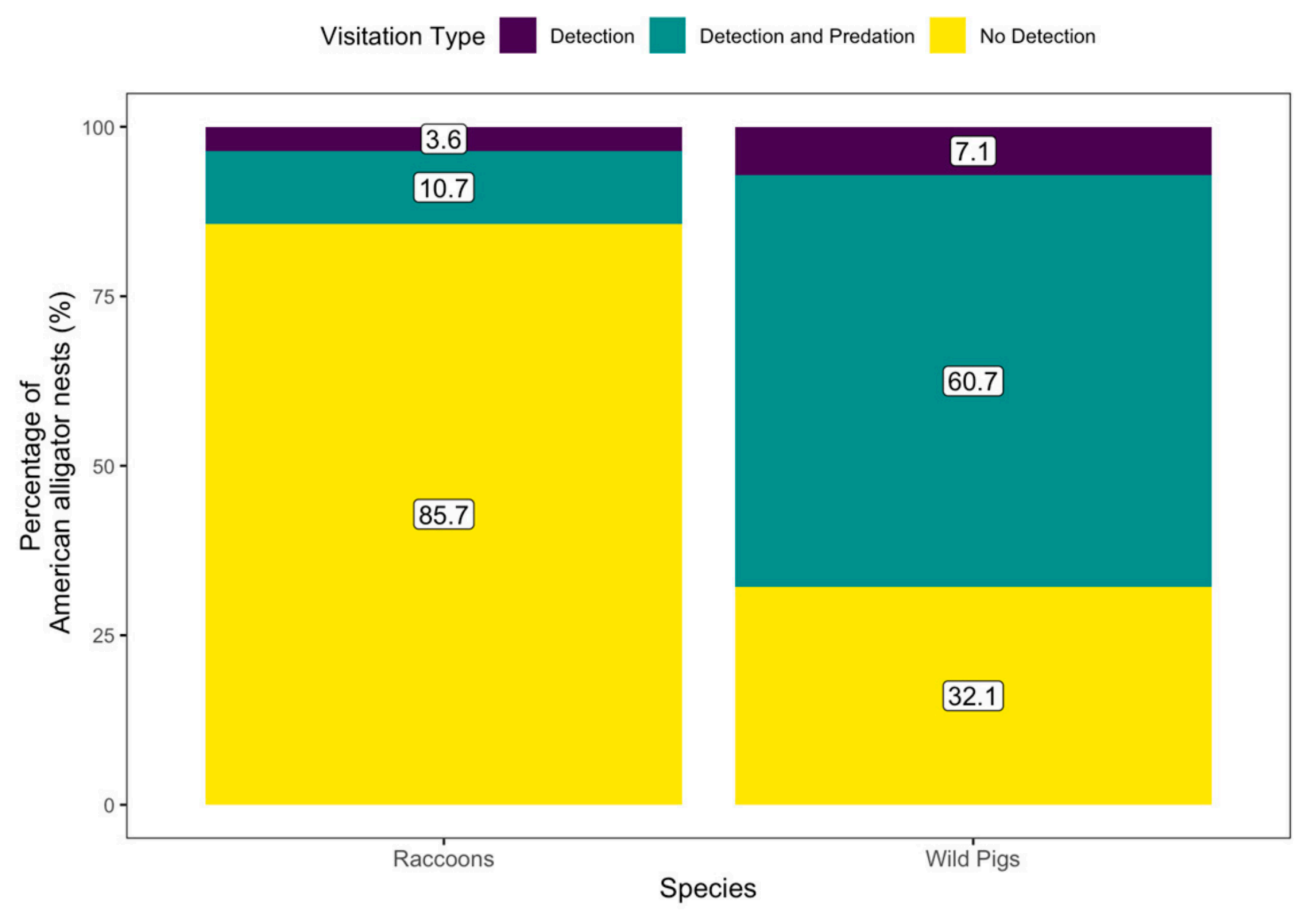
Stacked bar chart of raccoon and wild pig visitation and predation at *American alligator* nests. Data were generated from camera data collected from 28 alligator nests in the J.D. Murphree Wildlife Management Area during the summer of 2022 and 2023 in Jefferson County, Texas.

Across 2022 and 2023, we recorded two bobcats, one coyote (*Canis latrans*), one river otter, eastern cottontails (*Sylvilagus floridanus*), fire ants (*Solenopsis* spp.), various bird species (e.g., red-winged black birds [*Agelaius phoeniceus*]), raccoons and wild pigs at alligator nests, where only the latter two species were observed depredating alligator nests. We observed wild pigs at 19 out of 28 alligator nests (67.8%). Seventeen of those 19 nests (89.5%) were predated by the wild pigs (Figure 2). We also observed raccoons at four of the 28 nests (14.3%), three of which they depredated (75.0%; Figure 2). At these four nest sites, both wild pig and raccoon presence overlapped, where 50% of nests were depredated by both species, 25% of the nests were depredated solely by wild pigs, and the remaining 25% were depredated by raccoons. At all nest sites where both species overlapped, we recorded wild pig presence first, with raccoons appearing in our images 2–22 days after wild pigs. The odds of visiting alligator nests were 11.95 times higher for wild pigs than raccoons (Fisher’s exact test *p* < 0.01) where the odds of foraging were 9.91 times higher for wild pigs than raccoons (Fisher’s exact test *p* = 0.02). We recorded seven nest sites where hatchling liberation occurred, despite depredation by wild pigs, with one nest site where both wild pig and raccoon depredation occurred. We observed attending female alligators defending at five nest sites. There were two nest sites (7.1%) with no evidence of hatchling liberation or depredation events.

### 3.2. Variation Within Continuous Landscape Metrics

**Figure 3:**
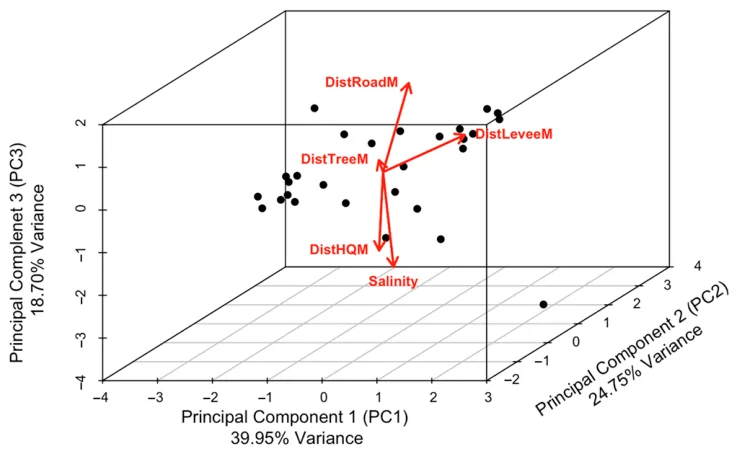
Biplot of the first three principal components generated from camera data collected from 28 alligator nests in the J.D. Murphree Wildlife Management Area during the summer of 2022 and 2023 in Jefferson County, Texas. The axes (bottom principal component 1 [PC1], right principal component 2 [PC2], and left principal component 3 [PC3]) represent numerical PCA scores that correspond with each alligator nest (i.e., displayed black dots). Principal component 1 has strong negative influence from distance to headquarters (DistHQM) and distance to road (DistRoadM). Conversely, PC2 has strong positive influence from DistRoadM and distance to the nearest tree (DistTreeM).

The PCA revealed that principal component 1 (PC1) explained approximately 39.95% of the variance, while principal component 2 (PC2) accounted for 24.75% of the variance and principal component 3 (PC3) accounted for 18.70% of the variance (see Figure 3). Together, the first two components accounted for approximately 64.7% of the total variance in the dataset; therefore, we proceeded with our analyses using PC1 and PC2. Notably, PC1 was dominated by variables with high negative loadings, particularly distance to trees (DistTreeM: −0.576) and distance to headquarters (DistHQM: −0.574). Conversely, PC2 was characterized by a strong positive loading from distance to road (DistRoadM: 0.748).

### 3.3. Model Selection and Significant Predictors

When performing the backwards elimination process, our global models, and the subsequent models assembled, failed to converge. Therefore, we made inference only on models assembled using the forward selection. In total, we assembled eight logistic regression models. Our best performing model, Model 3, revealed an effect for distance to roads (β = −0.01, *p* = 0.05) and distance to headquarters (β = 0.0004, *p* = 0.09), though this did not reach statistical significance. Model 3 was the top ranked model yielding an AICc value of 21.67 and a weight of 0.58 (Table 1). To evaluate the fit of the selected models, we applied the Hosmer–Lemeshow goodness-of-fit test. For our top model, the test statistic was χ^2^ = 2.15 (df = 8, *p* = 0.98).

## 4. Discussion

This study investigated various landscape factors influencing wild pig predation on alligator nests, with a particular focus on nest location, wetland type, soil type, landcover, distance to roads, distance to headquarters, distance to levees, distance to trees, and mean summer salinity levels. We constructed our models referencing a study predicting wild pig damage in Boso Penninsula, Japan, where Saito et al. [60] found the best model incorporated both environmental factors (distance from forest edges, roads, and rivers) and countermeasures (electric fencing and weeding), demonstrating that a combination of factors is crucial for predicting and mitigating damage. Despite this, our findings indicate the effect of anthropogenic features on wild pig predation risk on alligator nests.

Our results indicate a significant negative relationship between distance to roads and the likelihood of wild pig predation on alligator nests. As the distance to roads increases, the likelihood of predation decreases, suggesting that proximity to roads may increase the risk of predation. This finding is consistent with the idea that disturbances associated with roads could create habitats that facilitate wild pig movement, increasing their accessibility to alligator nests. For example, Lendrum et al. [65] conducted a study on the influence of anthropogenic disturbance on the predation risk of large carnivores on mule deer (*Odocoileus hemionus*) and found that pipelines and well pads may have impeded predation while proximity to roads created a greater risk for predation. Closer proximity to anthropogenic features like roads and trails may lead to an increase in predation risks because predators utilize these features as travel routes, especially during times of low human activity [65,66,67]. In Patagonia, Argentina, Gantchoff and Belant [68] found that, although wild pigs avoided areas of human settlement, they often utilized roads presumably as movement corridors. It is important to note that the roads present in the Murphree WMA are not main roads, they are secondary roads closed off to the public, infrequently traveled and largely unimproved (i.e., bare dirt or grass). In contrast, there have been numerous studies on crop damage that suggest wild pigs actively avoid roads [60,69,70,71], of which the roads are in similar condition to the roads in the Murphree WMA. It is possible there are confounding factors in these studies, such as risk of being killed by humans, that may drive wild pigs to avoid roads, or the type of road surface (e.g., paved roads). Nevertheless, while we did not measure wild pig movement in our study area, we saw clear evidence that closer proximity roads may increase the likelihood of wild pig predation on alligator nests.

In addition to distance to roads, other anthropogenic features may contribute to alligator nest predation by wild pigs and other predators. Our analysis identified distance to headquarters (i.e., human infrastructure) posed a small but not statistically significant influence on predation risk. The positive association between wild pig predation and proximity to headquarters suggests that nests located further from these areas are at a higher risk of wild pig predation. This might be attributed to increased human activity (particularly airboat and heavy equipment operations at or near headquarters), which might deter wild pigs or alter their behavior in a way that reduces the likelihood of them encountering alligator nests, further emphasizing the influence of anthropogenic factors on wildlife behavior and predation dynamics. We suspect that wild pigs in this system, where they are intensively persecuted by humans, are wary of being too close to areas with regular human activity.

To see how differences in the natural habitat affect predation, we included distance to trees, levees, wetland type, landcover type, nest location characteristics, soil type and summer salinity. While these variables did not show a significant effect on predation risk, the inclusion of these variables, especially salinity, still highlights the importance of understanding habitat characteristics. The primary difference between estuarine and palustrine wetlands lies in their salinity levels and tidal influence [72]. Estuarine wetlands are more saline and subject to tidal influences, which shapes a vegetation community adapted to saline conditions and frequent flooding [73]. Palustrine wetlands, being freshwater systems, support vegetation that is not adapted to high salinity, often resulting in more diverse species that thrive in non-saline nontidal environments [74]. Howes et al. [75] and Alldred et al. [76] found that marshes with high salinity levels generate more root biomass compared with adjacent low-salinity marshes. Salinity levels within these wetlands impact vegetation structure and diversity, which may indirectly influence wild pig presence and foraging [47,48]. Previous studies documented wild pigs in salt marshes searching for roots, tubers, and invertebrates [49,50]. Additionally, alligator nest success and potential predation risk can be attributed to nest location characteristics (where, in our study, we defined these locations as levees, banks, or open water). Joanen and McNease [26] note that high water levels reduce predation risk by raccoons, and thus low water levels or dry nesting seasons pose higher predation risk. While these variables (e.g., salinity levels, wetland type, landcover type, or nest location characteristics) were not significant in our top performing model, we suggest that future studies continue to consider impacts of landscape metrics on wild pig damage in wetlands, as, for example, salinity levels may vary across wetland types and seasonal changes.

Given the relationship between habitat characteristics and wild pig foraging behavior, it is important to note that nest predation does not necessarily indicate nest failure. Interestingly, seven nests predated by wild pigs still had evidence of hatchling liberation. Alligator nest success after predation has been seen before in Georgia [37] and South Carolina [34]. Ruckel and Steele [37] located one nest partially predated by racoons, but the mother repaired the nest and the rest of the eggs eventually hatched. They recorded a second nest partially predated by raccoons but resulted in hatchling liberation due to human involvement by covering the remaining eggs. Rainwater et al. [34] also witnessed hatchling liberation at two nests that were previously subjected to nest predation by Virginia opossums. With nest predation events occurring several times before and after hatchling liberation it would seem plausible that wild pigs could be foraging the nests for remains like infertile eggs or eggshells. These findings underscore the resilience of alligator nesting behavior in the face of predation and the complexity of predator–prey interactions. As such, they highlight the need for further research to explore the dynamics of nest predation and its implications for alligator conservation. To be succinct, not every damaged nest by a predator means that all, or any, hatchlings are lost. To better understand the detailed effects of wild pig damage to alligator nests and ultimately the species’ conservation, future studies should examine the rates of hatching liberation and survival in the face of wild pig damage to nests.

In this study, we utilize the American alligator as an example to investigate the effects of wild pig nest predation on ground nesting herpetofauna. Various crocodilian species are also affected by wild pig nest depredation, whether in their introduced ranges (e.g., freshwater crocodile [*Crocodylus johnstoni*] in Australia [77], Pantanal caiman [*Caiman crocodilus*] in Brazil [78], broad-snouted caiman [*Caiman latirostris*] in Argentina [79]) or native ranges (e.g., Chinese alligator [*Alligator sinensis*] in China [80]). Wild pig damage can also lead to degradation of nesting habitat, as agencies expressed concern with the saltwater crocodiles (*Crocodylus porosus*) in Australia [81], or pose risk of disease transfer (e.g., *Trichinella* spp. in Papua New Guinea [82]). While some species (e.g., the saltwater crocodile) will readily consume wild pigs [81], nest disturbance attributed to the species may have long-lasting effects. It is well known that nest success and hatchling sex ratio are dependent on temperature which is determined by nest insulation, nest location, and additional abiotic factors [26,83,84]. For example, sensitive species such as the critically endangered Chinese alligator [85] are dependent on nest success for species recovery. The additive disturbance of wild pig damage to Chinese alligator nests could affect hatchling sex ratio, and we surmise this would result in the production of more males than females as females are produced at low temperatures (i.e., edges of the nest) while males are produced at higher temperatures (i.e., core of the nest), similar to the American alligator [26,84]. While hatchling liberation post predation event can be considered nesting success, the production of mostly male offspring into a critically endangered population does not yield long-term benefit to the population. To be clear, understanding similarities between the two species (i.e., Chinese alligator and American alligator) or others and nest predation risks can help guide conservation and management efforts to mitigate future loss.

### Study Limitations

Although our selected models are robust in explaining the observed patterns of wild pig predation on alligator nests, the relatively low sample size (i.e., sampling 28 nests across two years with only 17 wild pig predation events and 4 raccoon predation events) and potential confounding variables indicate the need for caution in generalizing these findings. Additionally, we recognize the implications from this study regarding distance to road effects on wild pig predation risk would greatly benefit from a study monitoring wild pig movements relative to alligator nest locations. We recommend that future research should aim to include larger datasets and take measurements of each nest’s microclimate, including soil samples, salinity samples, and canopy cover, and wet globe temperature.

## 5. Conclusions

Wild pigs are reported as an avifauna nest predator. Yet, to our knowledge, no study has quantified wild pig predation on American alligator nests, a large nesting herpetofauna that was previously listed as an endangered species in the United States from 1966 to 1987. The objective of our study was to quantify wild pig predation on alligator nests in Texas coastal marshes between 2022 and 2023 and determine the relationship between landscape characteristics (nest location, wetland type, soil type, landcover, distance to roads, distance to management headquarters [i.e., human infrastructure], distance to levees, distance to trees, and salinity levels) and wild pig nest predation. At 28 alligator nests, we documented 17 instances of wild pig nest predation, where distance to roads negatively correlated with wild pig nest predation (β = −0.01, *p* = 0.05). We recognize that our relatively low sample size (28 nests and only 17 predation events) limits our ability to generalize our findings, yet our results still indicate an effect of distance to roads on predation risk, as seen by other studies with various taxa. As anthropogenic influences, such as road proximity, appear to increase the risk of alligator nest predation by wild pigs, habitat management strategies should prioritize minimizing impact on native fauna. Therefore, implementing targeted control measures in these areas could enhance nest survival rates by taking advantage of the accessibility for management, while simultaneously mitigating the increased predation risk posed by wild pigs’ easy access to these locations. Additionally, increasing awareness among land managers and stakeholders about the interplay between human infrastructure and wildlife dynamics could foster more effective conservation practices. We suggest that future research incorporates a larger sample size, along with additional metrics such as canopy cover and wet globe temperature and their effects on wild pig predation risk on alligator nests.

## Figures and Tables

**Table 1:** AICc model output of the logistic regression models for the relationship between the predictor variables (distance to roads [m; DistRoadM], distance to headquarters [m; DistHQM], distance to levee [m; DistLeveeM], distance to tree [m; DistTreeM], mean summer salinity [ppt; Salinity], nest location, soil, wetland, and landcover type, and principal component 1 [PC1] and principal component 2 [PC2] generated from our principal component analysis) and wild pig predation on alligator nests. Models were generated from camera data collected from 28 alligator nests in the J.D. Murphree Wildlife Management Area during the summer of 2022 and 2023 in Jefferson County, Texas.

Model	Predictor Variables	AICc	LogLik	df	ΔAICc	AICcWt
3	DistRoadM + DistHQM + DistLeveeM	21.67	−5.97	4	0.00	0.59
4	PC2	24.03	−9.78	2	2.34	0.18
2	DistRoadM + DistHQM	24.17	−8.59	3	2.50	0.17
1	DistRoadM	27.35	−1.14	2	5.68	0.03
5	PC2 + Landcover	27.49	−3.94	7	5.81	0.03
Null		39.67	−1.88	1	18.00	0.00
PCA Global	PC1 + PC2 + Nest Location + Soil + Wetland + Landcover	70.00	<−0.01	15	48.33	0.00
Global	DistRoadM + DistHQM + DistLeveeM + DistTreeM + Nest Location + Soil + Wetland + Landcover	112.00	<−0.01	18	90.33	0.00

## Data Availability

Data supporting the findings of this study are available from the corresponding author upon reasonable request.
